# Longitudinal Retinal and Cervical Spinal Cord Changes in Multiple Sclerosis: Associations with Clinical, Cognitive, and Motor Measures

**DOI:** 10.3390/jcm15218491

**Published:** 2026-11-01

**Authors:** Bedirhan Menguc, Emine Rabia Koc, Gamze Ucan Gunduz, Rifat Ozpar, Furkan Saridas, Guven Ozkaya

**Affiliations:** 1 Department of Neurology, Faculty of Medicine, Bursa Uludağ University, 16059 Bursa, Türkiye; 2 Department of Ophthalmology, Faculty of Medicine, Bursa Uludağ University, 16059 Bursa, Türkiye; 3 Department of Radiology, Faculty of Medicine, Bursa Uludağ University, 16059 Bursa, Türkiye; 4 Department of Biostatistics, Faculty of Medicine, Bursa Uludağ University, 16059 Bursa, Türkiye

**Keywords:** multiple sclerosis, spinal cord, tomography, optical coherence

## Abstract

**Background/Objectives:** Multiple sclerosis (MS) is a central nervous system disease that can cause severe disability. Identifying biomarkers associated with disease progression and disability is important for MS follow-up. Optical coherence tomography (OCT) parameters, measurements of cervical spinal atrophy, and cognitive and motor performance tests were evaluated along with their changes over one year to investigate their potential role in MS follow-up. **Methods:** This retrospective study included 79 patients with MS. OCT and cervical magnetic resonance imaging performed at 1-year intervals, along with cognitive and motor performance tests, were evaluated. Cervical mean cross-sectional area (mCSA) was used to assess spinal atrophy. **Results:** Over one year, cervical mCSA and ganglion cell–inner plexiform layer (GCIPL) measurements significantly decreased. Their annual changes were not significantly correlated in the Pearson analysis. The age-, sex-, and disease-duration-adjusted linear analysis likewise did not identify a statistically significant association. **Conclusions:** Cervical mCSA and GCIPL thickness significantly decreased over one year, but their changes were not significantly correlated. These findings suggest that cervical mCSA and GCIPL may provide complementary information on longitudinal structural changes in MS. However, because measurement reproducibility and absolute error were not quantified for the single-reader cervical measurements, the observed mCSA decrease should be interpreted cautiously, as the extent to which it reflects biological change rather than measurement variability remains uncertain. The clinical relevance of these findings requires further validation.

## 1. Introduction

Multiple sclerosis (MS) is an inflammatory, demyelinating, and neurodegenerative disease of the central nervous system (CNS) that can lead to neurological disability. The clinical course of MS can manifest as either relapsing-remitting or progressive forms [1]. Neurodegenerative processes become more prominent during the progressive phase of the disease [2].

The early and reliable identification of biomarkers that may contribute to the assessment of disease progression in MS is critically important for guiding appropriate treatment strategies among the growing number of therapeutic options available today and for preventing irreversible neurological damage. Therefore, there is an increasing need for biomarkers that reliably reflect the underlying processes associated with disease progression. In this context, various methods are used to assess neurodegeneration and monitor disease progression in MS.

Optical coherence tomography (OCT) is a non-invasive imaging technique that enables the evaluation of retinal layers. Thinning of the retinal nerve fiber layer (RNFL) and ganglion cell–inner plexiform layer (GCIPL) observed in patients with MS is considered a structural marker of neuroaxonal damage and may reflect neurodegenerative processes occurring in the CNS [3].

In a study conducted by Vidal-Jordana et al., RNFL and GCIPL thicknesses in patients with MS were found to be significantly associated with both cerebral and spinal cord atrophy. Furthermore, these OCT-derived retinal measures appeared to be more closely associated with MS-related disability than magnetic resonance imaging (MRI) measures [4]. These findings suggest that OCT may be useful for monitoring the neurodegenerative process and assessing disease progression in patients with MS.

Cervical spinal cord atrophy has emerged as an important biomarker associated with MS progression. Reductions in spinal cord cross-sectional area (CSA) can be measured using MRI, and these measures are considered objective indicators of disease burden [5]. Several studies have supported the role of spinal cord atrophy in MS progression, including progression independent of relapses [6]. Therefore, the early assessment of cervical spinal cord atrophy may provide valuable information for identifying patients at risk of disease progression.

MS can also affect cognitive function, and cognitive impairment may develop independently of relapses [7]. In addition, various clinical assessment tools are used to evaluate motor performance and monitor patients’ functional status and disease progression [8,9].

The aim of this study was to evaluate longitudinal changes in retinal layer thickness and cervical spinal cord CSA and to investigate the associations of these structural measures with clinical, cognitive, and motor outcomes in people with MS.

## 2. Methodology

### 2.1. Patient Selection and Clinical Evaluation

This retrospective study included 79 patients with MS who were regularly followed at the MS Outpatient Clinic of the Department of Neurology, Faculty of Medicine, Uludağ University, Bursa, Türkiye. All patients had been diagnosed with MS according to the 2017 Revised McDonald criteria, which were applicable at the time of diagnosis and ethical approval.

Patients aged between 18 and 65 years were eligible for inclusion. Clinical and imaging data were retrospectively obtained from the patients’ medical records. The evaluated parameters included OCT measurements of the RNFL and GCIPL, cognitive assessments using the Montreal Cognitive Assessment (MoCA) and Symbol Digit Modalities Test (SDMT), and motor performance assessments using the Timed 25-Foot Walk (T25-FW) and 9-Hole Peg Test (9-HPT). Cervical MRI scans of sufficient quality to allow measurement of the cervical spinal cord CSA were also evaluated.

Eligibility for individual analyses depended on the availability and validity of the relevant data. For the OCT analyses, patients with a history of bilateral optic neuritis (ON) were excluded. Patients with missing or invalid data for a specific parameter were excluded only from the corresponding analysis. In addition, patients with another organic or hereditary disease that could lead to spinal cord atrophy were not included in the relevant spinal cord analyses.

A total of 79 patients were retrospectively screened. Ten patients were excluded from the RNFL and GCIPL analyses because of a history of bilateral ON (*n* = 4) or missing OCT data (*n* = 6). In addition, three patients were excluded from the T25-FW analysis, five from the SDMT analysis, three from the MoCA analysis, and one from the 9-HPT analysis because they were unable to complete the respective tests. No participant was excluded from all analyses. Thus, participants with available and valid data were included in the relevant analyses, whereas missing or invalid data were handled on an analysis-specific basis.

Demographic characteristics, disease duration, disease-modifying therapy (DMT), clinical examination findings, Expanded Disability Status Scale (EDSS) scores, T25-FW and 9-HPT performance, MoCA and SDMT scores, and OCT measurements of RNFL and GCIPL were retrospectively obtained from the patients’ medical records. Cervical MRI scans obtained at baseline and at the 1-year follow-up were also reviewed. CSA measurements were performed at each cervical level, and the mean cross-sectional area (mCSA) was calculated.

### 2.2. Ethics Approval

This study was conducted after being deemed ethically acceptable in accordance with Decision No. 2024-15/2 dated 25 September 2024, of the Ethics Committee for Health Research at the Faculty of Medicine, Bursa Uludağ University.

### 2.3. Cognitive and Motor Performance Assessments

The EDSS was used for clinical assessment, the MoCA and SDMT for cognitive assessment, and the T25-FW and 9-HPT for motor performance assessment. Data from these tests, which assess disability level, global cognitive function, information processing speed, walking performance, and upper extremity motor skills, respectively, were analyzed using recorded data from baseline and 12-month follow-up assessments.

### 2.4. Optical Coherence Tomography

In this study, OCT examinations performed by the Department of Ophthalmology as part of the clinical evaluation of patients with MS were retrospectively reviewed to assess retinal structural changes. The thicknesses of the RNFL and GCIPL were evaluated.

OCT images were acquired using a spectral-domain OCT device (Spectralis HRA+OCT; Heidelberg Engineering, Heidelberg, Germany). All measurements were performed without cycloplegia.

For macular analysis, the Early Treatment Diabetic Retinopathy Study (ETDRS) grid, consisting of concentric circles with diameters of 1, 3, and 6 mm centered on the fovea, was used. Macular scans were centered on the fovea, and the device’s real-time eye-tracking technology (TruTrack; Heidelberg Engineering, Heidelberg, Germany) was used to maintain scan alignment during image acquisition. Measurements were obtained from the parafoveal sectors (superior, inferior, nasal, and temporal) located within the inner ETDRS ring (1–3 mm). The OCT software(Heidelberg Eye Explorer (version 1.9.17.0; Heidelberg Engineering, Heidelberg, Germany) automatically provided separate thickness measurements for the ganglion cell layer (GCL) and inner plexiform layer (IPL). As the software did not provide a combined GCIPL measurement, GCIPL thickness was calculated by summing the corresponding GCL and IPL thicknesses for each parafoveal sector. The arithmetic mean of these four sectoral GCIPL values was then calculated for each visit and used in the longitudinal analyses.

For peripapillary RNFL analysis, a circular scan pattern (3.5 mm in diameter, comprising 768 A-scans) was used. RNFL thickness was automatically segmented using the same software, and the global RNFL thickness was recorded and used for longitudinal analyses. All measurements were recorded in micrometers (µm).

Only well-centered OCT scans with an image quality score of ≥25 dB were included in the analysis. All automated retinal layer segmentations were visually inspected by an investigator. Scans with segmentation errors, insufficient image quality, or inadequate foveal centration were excluded from the analysis without manual correction. As this was a retrospective study, excluded scans were not repeated. The examiner responsible for collecting OCT measurement data from previously acquired scans was masked to the patients’ neurological status and EDSS scores.

In patients with a history of unilateral ON, the eye without a history of ON was included in the analysis. In patients without a history of ON, one eye was randomly selected for analysis. Patients with a history of bilateral ON were excluded from the OCT analyses. The same eye was assessed at both visits for each patient to ensure consistency in longitudinal comparisons.

### 2.5. Cervical Magnetic Resonance Imaging

All MRI examinations were performed using either a 3T scanner (Achieva Tx dStream^®^, Philips, Best, The Netherlands) or a 1.5T scanner (Aera^®^, Siemens, Erlangen, Germany). Although two different MRI scanners were used, baseline and 12-month cervical MRI examinations were acquired using the same scanner for each participant.

**Figure 1:**
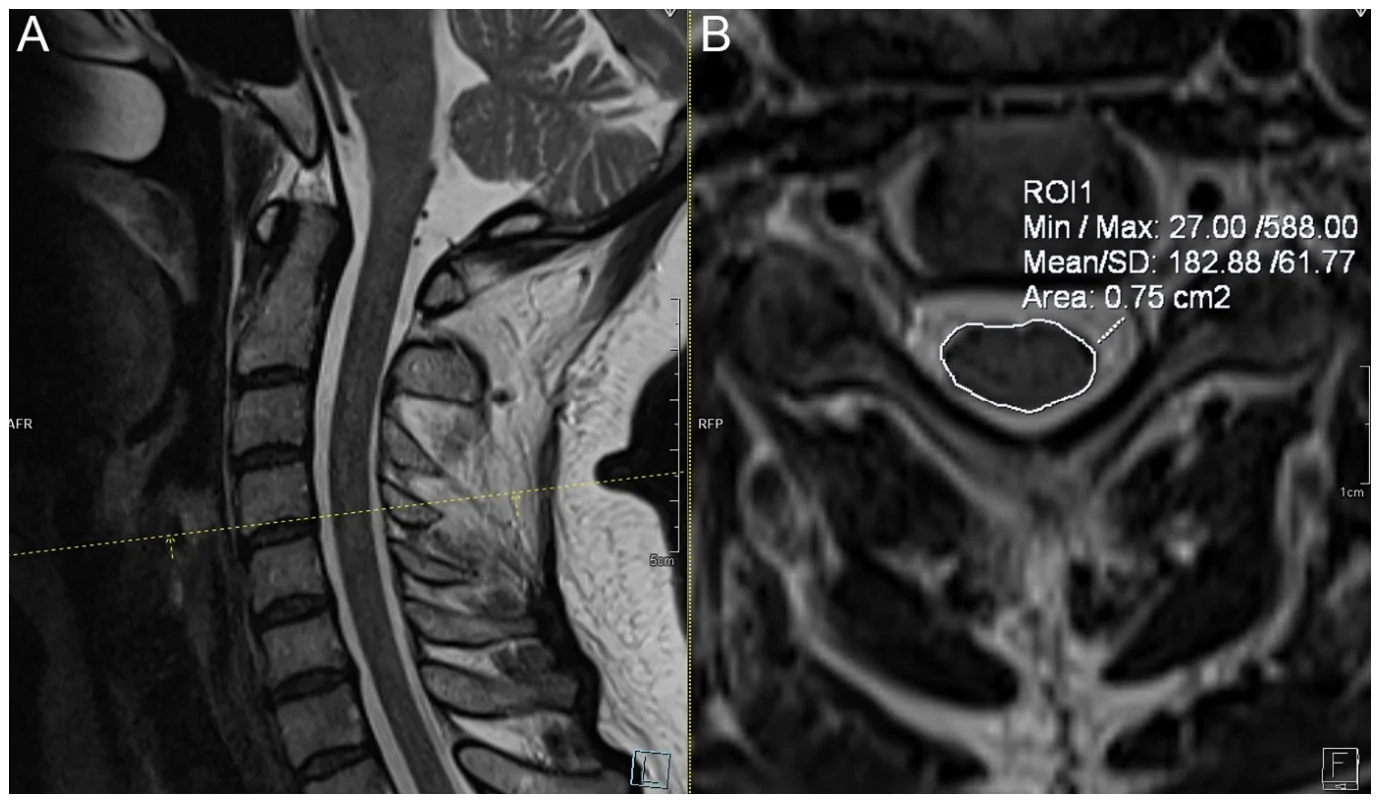
Manual cervical spinal cord cross-sectional area (CSA) measurement at the C4–C5 intervertebral level. (**A**) Sagittal T2-weighted image showing the location of the axial measurement plane. (**B**) Corresponding axial T2-weighted image showing the manually delineated spinal cord boundary used for CSA measurement. Scale bars and anatomical orientation markers are displayed in both panels.

For spinal cord CSA measurements, contiguous axial T2-weighted turbo spin-echo (TSE) images with a voxel size of 0.7 × 0.85 × 3 mm were used. The spinal cord boundary was manually delineated on axial T2-weighted images at six intervertebral levels (C2–C3, C3–C4, C4–C5, C5–C6, C6–C7, and C7–T1), and the CSA at each level was calculated in mm^2^ using the area measurement tool of the imaging software. No dedicated or automated spinal cord segmentation software was used. The mCSA was calculated as the arithmetic mean of the CSA measurements obtained at these six levels and was used in the analyses. An example of the manual CSA measurement at the C4–C5 level is shown in Figure 1.

All cervical spinal cord CSA measurements were performed manually by a single neuroradiologist with 10 years of experience in neuroradiology. The reader was blinded to clinical information, examination timepoint (baseline or one-year follow-up), and scanner field strength. At both visits, axial sections were acquired at the intervertebral disc levels, perpendicular to the local longitudinal axis of the spinal cord. Measurements were obtained at the six intervertebral levels specified above. Each examination was measured once; no repeated measurement sessions or independent assessments by a second reader were performed.

### 2.6. Statistical Analysis

The distribution of continuous variables was assessed using the Shapiro–Wilk test. Continuous variables were summarized as mean ± standard deviation or median (interquartile range), as appropriate, and categorical variables as frequencies and percentages. Longitudinal changes between baseline and 12 months were evaluated using the Wilcoxon signed-rank test in participants with paired measurements. Within-person changes were calculated as the 12-month value minus the baseline value and reported with 95% confidence intervals (CIs) obtained by bootstrap resampling (5000 iterations).

Associations among clinical, cognitive, and imaging parameters were assessed using Pearson’s product–moment correlation coefficients, with participant-bootstrap 95% confidence intervals (1500 resamples). Three main comparisons were evaluated: longitudinal changes in cervical mCSA and GCIPL thickness, and the association between their annual changes; Holm adjustment was applied to this family. The remaining correlations were exploratory, with Benjamini–Hochberg false discovery rate (BH-FDR) adjustment applied separately to the 28 pairwise comparisons at baseline, at 12 months, and for annual changes. Distributional characteristics and influential observations were considered when interpreting the Pearson correlations.

Multivariable linear regression analyses examined the association of annual GCIPL change with changes in cervical mCSA, the 9-HPT, and EDSS, adjusting for age, sex, and disease duration. Heteroskedasticity-consistent (HC3) standard errors were used to calculate regression coefficient confidence intervals and *p*-values. Model diagnostics included an assessment of influential observations.

Additional sensitivity analyses examined the consistency of cervical CSA changes across the six anatomical levels. Annual changes were evaluated separately for each level, and leave-one-level-out analyses were conducted by recalculating mean CSA after sequentially excluding each cervical level. Associations between these alternative CSA measures and GCIPL change were assessed using Pearson correlation. Individual GCIPL and mCSA trajectories were also visualized.

All statistical tests were two-sided, with statistical significance defined as *p* < 0.05 after multiplicity adjustment where applicable. The initial statistical analyses were conducted using IBM SPSS 29.0.2.0 (IBM Corp. Released 2023. IBM SPSS Statistics for Windows, Version 29.0.2.0 Armonk, NY, USA: IBM Corp).

## 3. Results

A total of 79 individuals with MS were included in the study, all of whom had relapsing-remitting multiple sclerosis (RRMS). The demographic and clinical characteristics of the study population are presented in the table (Table 1).

At the one-year follow-up, GCIPL thickness and cervical spinal cord CSA measures, including mCSA and CSA measurements at the evaluated cervical levels, were significantly lower than at baseline. T25-FW values also differed significantly between baseline and the one-year follow-up, whereas no significant changes were observed in EDSS, 9-HPT, SDMT, MoCA, RNFL thickness, or cervical spinal cord T2 lesion count (Table 2).

Annual CSA changes were negative at all six cervical levels (median change, −0.820 to −1.040 mm^2^), with reductions observed in 67/79 (84.8%) to 78/79 (98.7%) participants by level. In exploratory leave-one-level-out analyses, median changes in the alternative five-level mean CSA remained negative (−0.870 to −0.928 mm^2^). Correlations between these alternative indices and GCIPL change were weak and not statistically significant (Pearson r = 0.048–0.143; all *p* ≥ 0.241; Supplementary Table S1).

At baseline, 19 of 28 exploratory Pearson correlations remained statistically significant after BH-FDR adjustment. Cervical mCSA was positively correlated with GCIPL thickness (r = 0.265, *n* = 69, bootstrap 95% CI 0.050 to 0.459; adjusted *p* = 0.043; Table 3).

At 12 months, 18 of 28 exploratory Pearson correlations remained statistically significant after BH-FDR adjustment. The GCIPL–mCSA correlation was positive but did not remain significant after adjustment (r = 0.247, *n* = 69, bootstrap 95% CI 0.053 to 0.438; adjusted *p* = 0.059; Table 4).

**Figure 2:**
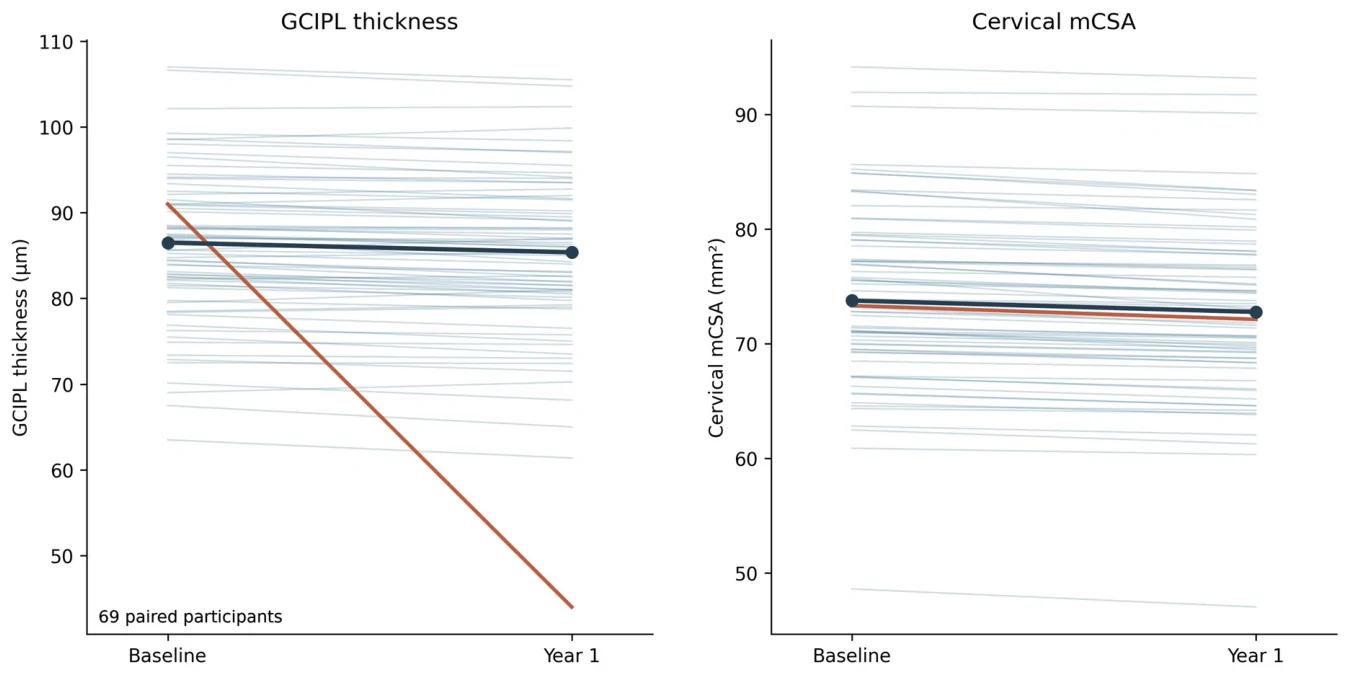
Individual longitudinal trajectories of GCIPL thickness and cervical mCSA from baseline to year 1 among participants with paired data for both measures (*n* = 69). Thin blue lines represent individual patient trajectories connecting baseline and one-year measurements. The red line highlights the trajectory of the same patient in both panels, while the thick black line with circular markers represents the group medians at the two time points.

None of the 28 exploratory Pearson correlations between annual changes remained statistically significant after BH-FDR adjustment. The association between annual GCIPL and cervical mCSA changes was r = 0.122 (*n* = 69; adjusted *p* = 0.772), whereas the GCIPL–9-HPT change correlation was r = −0.289 (*n* = 68; adjusted *p* = 0.206; Table 5). Individual longitudinal trajectories are shown in Figure 2.

### Exploratory Partially Adjusted Change-Score Analyses

Age-, sex-, and disease-duration-adjusted models did not show a statistically significant linear association between annual GCIPL change and changes in cervical mCSA, 9-HPT, or EDSS (Supplementary Table S2). These exploratory models did not include adjustment for MRI scanner field strength or DMT category.

In paired analyses, cervical mCSA decreased in 79 patients (median change −0.91 [−1.20 to −0.69] mm^2^; bootstrap 95% CI −1.01 to −0.80), and GCIPL decreased in 69 patients (median change −1.00 [−1.62 to −0.38] µm; bootstrap 95% CI −1.25 to −0.75). T25-FW showed a median paired decrease of 0.50 [−1.00 to 0.50] in 76 patients (95% bootstrap CI, −0.50 to 0.00). Paired sample sizes and change estimates for all parameters are presented in Table 2.

## 4. Discussion

In this study, we investigated the relationships between retinal layer thicknesses (RNFL and GCIPL) and cervical mCSA, a structural measure of cervical spinal cord atrophy, in patients with MS. We also evaluated one-year longitudinal changes in these structural measures and their associations with clinical, cognitive, and motor performance measures. Our findings show both similarities to and differences from those reported in previous studies.

Previous studies have suggested that cervical spinal cord atrophy is associated with disease progression in MS and may provide useful information for monitoring neurodegeneration [10,11]. Similarly, OCT-derived measures, particularly GCIPL and RNFL thicknesses, have been investigated as biomarkers reflecting neuroaxonal damage and disease progression in MS [12]. Several studies have also reported associations between retinal layer thickness and spinal cord atrophy, suggesting that these measures may reflect related aspects of neurodegeneration [4,13].

Our findings demonstrate a strong positive correlation between RNFL and GCIPL thicknesses. The relationship between GCIPL and cervical mCSA was weak but remained statistically significant at baseline after BH-FDR correction, whereas the corresponding association at year 1 did not. In a study conducted by Oh and colleagues, significant correlations were observed between RNFL measured by OCT and cervical CSA in patients with MS [13]. Although these findings suggest some cross-sectional association between retinal and spinal cord measures, they do not establish a shared mechanism or longitudinal coupling.

Annual changes in GCIPL and cervical mCSA were not significantly correlated in the Pearson analysis. Similarly, the age-, sex-, and disease-duration-adjusted linear model did not identify a statistically significant association. GCIPL thickness and cervical mCSA decreased significantly during follow-up, whereas no significant change was observed in RNFL thickness. The strong cross-sectional correlation between RNFL and GCIPL suggests that these measures provide related structural information; however, the different longitudinal patterns observed in our study may indicate differences in their sensitivity to short-term structural changes. Furthermore, the absence of a correlation between changes in GCIPL and cervical mCSA suggests that structural changes in these anatomically distinct regions may not occur in parallel over a one-year period. These findings do not establish biological independence between retinal and spinal cord changes. Individual trajectories are presented in Figure 2.

Cervical spinal cord atrophy has been reported in numerous studies as an indicator associated with disease progression in patients with MS [10,14]. In a single-center observational study by Bischof and colleagues, 360 patients with RRMS, 47 with secondary progressive MS (SPMS), and 80 matched healthy controls were evaluated during long-term follow-up. Over a 12-year follow-up period, patients with RRMS who converted to SPMS or exhibited silent progression were compared with those with stable RRMS and healthy controls. In patients who developed SPMS, faster cervical spinal cord atrophy was detected at least 4 years earlier than in the matched RRMS group. Additionally, silent progression and conversion to SPMS were associated with cervical spinal cord atrophy, suggesting that spinal cord atrophy may be detectable during earlier stages of the disease and may provide prognostic information regarding disease progression [14].

A study by Zeydan and colleagues demonstrated that cervical spinal cord atrophy can distinguish patients with early-stage progressive MS from those with other MS subgroups [10].

Given the increasing number of treatment options available, identifying structural changes before overt clinical progression remains an important research goal. In our study, single-reader cervical spinal cord CSA measurements were significantly lower at the one-year follow-up. However, statistical significance alone does not demonstrate that the observed difference exceeds measurement variability. Without study-specific reproducibility and absolute-error estimates, these findings cannot establish reliable detection of small annual changes in individual patients. Although the clinical significance of the observed changes remains uncertain, these findings may provide preliminary insights into longitudinal structural alterations in MS and warrant further investigation in larger studies using standardized acquisition protocols and repeated measurements.

It has been reported that cervical mCSA is negatively correlated with EDSS and that atrophy of the upper cervical spinal cord, in particular, is closely associated with clinical deterioration [15,16]. Our findings are broadly consistent with these observations, as cervical mCSA showed weak but significant negative correlations with EDSS and motor performance measures.

However, the results regarding retinal layer measurements show a more heterogeneous distribution in the literature. While some studies report that GCIPL and RNFL thicknesses are significantly associated with increasing disability [4], other studies have found no significant association between EDSS and GCIPL or RNFL [17]. Similarly, our findings did not demonstrate significant associations between RNFL or GCIPL thickness and EDSS, supporting the heterogeneous results reported in the literature.

Recent studies suggest that cervical spinal cord atrophy may be associated with clinical disability and motor performance. In a study by Morozumi and colleagues, cervical spinal cord gray matter CSA was identified as an important MRI measure associated with disability and sensorimotor dysfunction [15]. Although our study also identified significant associations between cervical mCSA and EDSS and motor performance measures, these associations were weak. This difference may stem from variations in patient populations, sample sizes, spinal cord measurement methods, and the timing of assessments. Furthermore, the inability to demonstrate similar relationships in the analyses of longitudinal changes suggests that changes in spinal cord measures and functional performance may not occur in parallel over a one-year period.

T25-FW duration decreased over one year (paired median change, −0.50; *n* = 76; Table 2), consistent with a shorter recorded walking time at follow-up. The magnitude of this change was modest, and its clinical relevance remains uncertain in a cohort with relatively low baseline disability. Variability in retrospective test administration and potential practice effects should be considered when interpreting this result.

In terms of cognitive performance, there are limited data directly examining the relationship between cervical spinal cord atrophy and cognitive measures. In our study, weak associations were found between cervical mCSA and cognitive measures. These relatively weak associations may reflect the fact that cognitive impairment in MS is influenced by multiple CNS regions and pathological processes, including supratentorial gray matter and thalamic pathology [18,19,20].

### Limitations

The use of CSA measurements rather than volumetric analyses in the assessment of the cervical spinal cord is one of the study’s limitations, as CSA provides more limited information regarding the overall extent of spinal cord atrophy. Nevertheless, CSA measurements remain among the commonly used approaches for assessing cervical spinal cord atrophy because of their relative feasibility [10,16]. In addition, the manual measurement approach and the absence of dedicated automated spinal cord segmentation software may have introduced measurement variability.

All cervical CSA measurements were performed once by a single neuroradiologist. Consequently, inter-rater agreement and intra-rater repeatability were not assessed, and study-specific absolute measurement error and the smallest detectable change were not quantified. Although the reader had 10 years of neuroradiology experience and was blinded to clinical information, examination timepoint, and scanner field strength, these precautions do not establish measurement reproducibility. Acquisition at the same intervertebral disc levels, perpendicular to the local longitudinal axis of the cord, and use of the same scanner for each participant at both visits helped standardize the measurements but did not eliminate possible variability from manual contouring, slice positioning, partial-volume effects, or residual differences in neck position and cord angulation. The use of two scanner platforms also introduced acquisition heterogeneity. Therefore, we cannot determine whether the small one-year differences exceed measurement error, and the observed mCSA decrease should not be interpreted as definitive evidence of biological atrophy or validated individual-level progression.

The inclusion of patients with a history of ON may also be considered a limitation. Although the eye without a history of ON was analyzed in patients with unilateral ON and patients with bilateral ON were excluded from the OCT analyses, a history of ON may still have influenced retinal measurements because subclinical or contralateral retinal changes cannot be completely excluded. It is known that patients with a history of ON exhibit greater atrophy in the contralateral eye compared to patients with no such history [21].

Other limitations of the study include the possibility that the one-year follow-up period may have been insufficient to reveal the clinical manifestations of neurodegenerative changes; the inability to fully control for confounding factors such as educational level in cognitive tests; and the wide range of age and disease duration within the study group, which may have contributed to variability in certain parameters. In addition, the absence of a healthy control group limits the interpretation of whether the observed longitudinal structural changes are specific to MS. Although the regression models were adjusted for age, sex, and disease duration, additional potential confounders, including MRI scanner field strength and disease-modifying treatment, could not be accounted for because the necessary patient-level information was unavailable.

Furthermore, quantitative measures of brain lesion burden and brain atrophy were not included, which may have limited the interpretation of the associations with cognitive measures.

Finally, although clinical measures were assessed longitudinally, confirmed disability progression was not specifically evaluated in this study. This limits the conclusions that can be drawn regarding the relationship between the observed structural changes and clinical disease progression.

## 5. Conclusions

In this study, OCT-derived retinal measures and cervical spinal cord MRI measurements were evaluated in patients with MS, together with clinical, cognitive, and motor performance measures. RNFL and GCIPL thicknesses were strongly correlated, while cervical mCSA showed weak associations with retinal measures and clinical and functional parameters. Single-reader cervical mCSA measurements and GCIPL thickness decreased over one year, but most annual changes in structural and clinical measures were not significantly correlated. The significant decrease in GCIPL thickness and the observed reduction in cervical mCSA measurements over one year suggest that these measures may have potential value in assessing longitudinal structural changes in MS. GCIPL may be sensitive to short-term longitudinal changes and, when interpreted alongside cervical mCSA and other clinical measures, may provide complementary information for monitoring structural changes associated with MS. Nevertheless, because cervical measurement reproducibility and absolute error were not quantified, the observed mCSA decrease should be interpreted cautiously as a potential indicator of biological tissue loss, and its reliability for detecting individual-level progression requires further validation. Larger prospective studies incorporating standardized longitudinal acquisition, independent and repeated measurements, and longer clinical follow-up are needed to establish the clinical relevance of these findings.

## Figures and Tables

**Table 1:** Demographic and Clinical Characteristics of the Study Population.

Variables		*n* (%)
Age		40.06 ± 10.57
Sex	Female	65 (82.3%)
	Male	14 (17.7%)
MS phenotype	RRMS	79 (100%)
DMT at baseline	Interferon	2 (2.5%)
	Glatiramer acetate	5 (6.3%)
	Teriflunomide	16 (20.3%)
	Dimethyl fumarate	23 (29.1%)
	Cladribine	7 (8.9%)
	Fingolimod	16 (20.3%)
	Alemtuzumab	6 (7.6%)
	No DMT	4 (5.1%)
Number of relapses		2.43 ± 2.39
Disease duration		6.96 ± 6.10

Descriptive statistics are presented as mean ± standard deviation (SD), frequency (*n*), and percentage (%). MS: Multiple sclerosis; RRMS: Relapsing-remitting multiple sclerosis; DMT: Disease-modifying therapy.

**Table 2:** Comparison of Baseline and One-Year Measurements.

Outcome (Paired *n*)	Baseline Median [IQR]	Year 1 Median [IQR]	Paired Δ Median [IQR]	Bootstrap 95% CI for Median Δ	Wilcoxon *p*
EDSS (*n* = 79)	1.00 [1.00–2.25]	1.00 [1.00–2.00]	0.00 [0.00–0.00]	0.00 to 0.00	0.226
9-HPT (s) (*n* = 78)	20.50 [18.50–22.50]	20.00 [18.50–22.00]	0.00 [−1.50–1.50]	−0.25 to 0.50	0.886
SDMT (*n* = 74)	32.00 [22.00–41.00]	32.00 [24.00–44.00]	0.50 [−3.00–4.75]	−1.50 to 2.00	0.274
MoCA (*n* = 76)	24.00 [22.00–26.00]	24.00 [21.75–27.00]	0.00 [−1.00–2.00]	0.00 to 1.00	0.081
RNFL (µm) (*n* = 69)	95.00 [88.00–103.00]	94.00 [87.00–101.00]	0.00 [−2.00–1.00]	−1.00 to 0.00	0.293
T25-FW (s) (*n* = 76)	6.50 [6.00–7.50]	6.00 [5.50–6.50]	−0.50 [−1.00–0.50]	−0.50 to 0.00	<0.001
GCIPL (µm) (*n* = 69)	86.50 [81.50–91.00]	85.38 [80.12–90.20]	−1.00 [−1.62–−0.38]	−1.25 to −0.75	<0.001
C2–C3 CSA (mm^2^) (*n* = 79)	78.10 [74.08–83.44]	77.34 [73.28–82.50]	−0.86 [−1.28–−0.39]	−1.02 to −0.70	<0.001
C3–C4 CSA (mm^2^) (*n* = 79)	79.59 [75.65–84.62]	79.00 [74.92–84.00]	−0.82 [−1.29–−0.21]	−0.99 to −0.62	<0.001
C4–C5 CSA (mm^2^) (*n* = 79)	80.75 [74.72–85.49]	79.94 [73.50–84.75]	−0.97 [−1.40–−0.39]	−1.09 to −0.66	<0.001
C5–C6 CSA (mm^2^) (*n* = 79)	76.92 [71.52–82.75]	76.32 [70.78–80.81]	−0.92 [−1.41–−0.36]	−1.17 to −0.62	<0.001
C6–C7 CSA (mm^2^) (*n* = 79)	70.17 [63.72–74.16]	69.08 [62.78–72.89]	−1.04 [−1.70–−0.44]	−1.25 to −0.83	<0.001
C7–T1 CSA (mm^2^) (*n* = 79)	57.83 [53.88–62.72]	57.40 [53.00–62.15]	−0.86 [−1.58–−0.11]	−1.03 to −0.60	<0.001
Cervical mCSA (mm^2^) (*n* = 79)	73.85 [69.74–78.79]	73.02 [68.99–77.77]	−0.91 [−1.20–−0.69]	−1.01 to −0.80	<0.001
Cervical T2 lesion count (*n* = 79)	2.00 [1.00–4.00]	2.00 [1.00–4.00]	0.00 [0.00–0.00]	0.00 to 0.00	0.680

*n* is the number of participants with both measurements; all descriptive summaries are computed within the paired subset. Δ = month 12 − baseline. Bootstrap percentile 95% confidence intervals quantify uncertainty in the median within-person change. EDSS: Expanded Disability Status Scale; 9-HPT: Nine-Hole Peg Test; SDMT: Symbol Digit Modalities Test; MoCA: Montreal Cognitive Assessment; RNFL: Retinal nerve fiber layer; T25-FW: Timed 25-Foot Walk; GCIPL: Ganglion cell–inner plexiform layer; CSA: Cross-sectional area; mCSA: Mean cross-sectional area; IQR: Interquartile range, CI: Confidence interval.

**Table 3:** Baseline Pearson correlations.

Pair (*n*)	Pearson r	Bootstrap 95% CI	Raw *p*	BH-FDR *p*
EDSS—T25-FW (*n* = 77)	0.669	0.426 to 0.798	<0.001	<0.001
EDSS—9-HPT (*n* = 78)	0.651	0.431 to 0.796	<0.001	<0.001
EDSS—SDMT (*n* = 75)	−0.452	−0.621 to −0.248	<0.001	<0.001
EDSS—MoCA (*n* = 76)	−0.260	−0.462 to −0.013	0.024	0.039
EDSS—RNFL (*n* = 69)	−0.088	−0.270 to 0.109	0.474	0.474
EDSS—GCIPL (*n* = 69)	−0.091	−0.305 to 0.145	0.456	0.473
EDSS—mCSA (*n* = 79)	−0.318	−0.511 to −0.103	0.004	0.009
T25-FW—9-HPT (*n* = 77)	0.703	0.418 to 0.857	<0.001	<0.001
T25-FW—SDMT (*n* = 73)	−0.555	−0.703 to −0.353	<0.001	<0.001
T25-FW—MoCA (*n* = 74)	−0.419	−0.632 to −0.106	<0.001	<0.001
T25-FW—RNFL (*n* = 68)	−0.155	−0.323 to 0.027	0.207	0.232
T25-FW—GCIPL (*n* = 68)	−0.191	−0.385 to 0.064	0.119	0.158
T25-FW—mCSA (*n* = 77)	−0.290	−0.542 to 0.071	0.011	0.020
9-HPT—SDMT (*n* = 74)	−0.628	−0.736 to −0.497	<0.001	<0.001
9-HPT—MoCA (*n* = 75)	−0.421	−0.602 to −0.186	<0.001	<0.001
9-HPT—RNFL (*n* = 68)	−0.132	−0.294 to 0.039	0.285	0.306
9-HPT—GCIPL (*n* = 68)	−0.204	−0.407 to 0.029	0.095	0.133
9-HPT—mCSA (*n* = 78)	−0.353	−0.587 to −0.085	0.002	0.004
SDMT—MoCA (*n* = 75)	0.708	0.597 to 0.793	<0.001	<0.001
SDMT—RNFL (*n* = 65)	0.184	−0.065 to 0.423	0.142	0.181
SDMT—GCIPL (*n* = 65)	0.336	0.100 to 0.544	0.006	0.013
SDMT—mCSA (*n* = 75)	0.150	−0.108 to 0.390	0.199	0.232
MoCA—RNFL (*n* = 66)	0.179	−0.055 to 0.382	0.149	0.182
MoCA—GCIPL (*n* = 66)	0.366	0.127 to 0.550	0.003	0.006
MoCA—mCSA (*n* = 76)	0.261	0.032 to 0.450	0.023	0.039
RNFL—GCIPL (*n* = 69)	0.743	0.613 to 0.844	<0.001	<0.001
RNFL—mCSA (*n* = 69)	0.259	0.048 to 0.451	0.032	0.047
GCIPL—mCSA (*n* = 69)	0.265	0.050 to 0.459	0.028	0.043

Pearson product–moment correlation; participant-bootstrap 95% confidence intervals (1500 resamples). BH-FDR adjustment was applied to the 28 correlations in this table. *n*: Number of participants included in each correlation analysis; CI: Confidence interval; BH-FDR: Benjamini–Hochberg false discovery rate; EDSS: Expanded Disability Status Scale; 9-HPT: Nine-Hole Peg Test; SDMT: Symbol Digit Modalities Test; MoCA: Montreal Cognitive Assessment; RNFL: Retinal nerve fiber layer; T25-FW: Timed 25-Foot Walk; GCIPL: Ganglion cell–inner plexiform layer; mCSA: Mean cross-sectional area.

**Table 4:** Pearson correlations at year 1.

Pair (*n*)	Pearson r	Bootstrap 95% CI	Raw *p*	BH-FDR *p*
EDSS—T25-FW (*n* = 76)	0.662	0.410 to 0.779	<0.001	<0.001
EDSS—9-HPT (*n* = 78)	0.626	0.360 to 0.769	<0.001	<0.001
EDSS—SDMT (*n* = 74)	−0.400	−0.573 to −0.177	<0.001	<0.001
EDSS—MoCA (*n* = 76)	−0.449	−0.615 to −0.263	<0.001	<0.001
EDSS—RNFL (*n* = 69)	−0.081	−0.259 to 0.135	0.508	0.548
EDSS—GCIPL (*n* = 69)	−0.076	−0.289 to 0.127	0.537	0.557
EDSS—mCSA (*n* = 79)	−0.333	−0.518 to −0.085	0.003	0.005
T25-FW—9-HPT (*n* = 76)	0.838	0.588 to 0.925	<0.001	<0.001
T25-FW—SDMT (*n* = 72)	−0.476	−0.620 to −0.277	<0.001	<0.001
T25-FW—MoCA (*n* = 73)	−0.515	−0.757 to −0.400	<0.001	<0.001
T25-FW—RNFL (*n* = 67)	−0.058	−0.198 to 0.182	0.641	0.641
T25-FW—GCIPL (*n* = 67)	−0.188	−0.346 to 0.047	0.128	0.171
T25-FW—mCSA (*n* = 76)	−0.436	−0.668 to −0.064	<0.001	<0.001
9-HPT—SDMT (*n* = 73)	−0.556	−0.680 to −0.404	<0.001	<0.001
9-HPT—MoCA (*n* = 75)	−0.593	−0.743 to −0.445	<0.001	<0.001
9-HPT—RNFL (*n* = 68)	−0.109	−0.269 to 0.103	0.376	0.421
9-HPT—GCIPL (*n* = 68)	−0.164	−0.361 to 0.098	0.182	0.232
9-HPT—mCSA (*n* = 78)	−0.359	−0.609 to 0.008	0.001	0.003
SDMT—MoCA (*n* = 74)	0.800	0.722 to 0.867	<0.001	<0.001
SDMT—RNFL (*n* = 65)	0.150	−0.087 to 0.362	0.234	0.285
SDMT—GCIPL (*n* = 65)	0.362	0.153 to 0.538	0.003	0.005
SDMT—mCSA (*n* = 74)	0.272	0.023 to 0.476	0.019	0.029
MoCA—RNFL (*n* = 66)	0.137	−0.083 to 0.337	0.272	0.317
MoCA—GCIPL (*n* = 66)	0.371	0.116 to 0.559	0.002	0.004
MoCA—mCSA (*n* = 76)	0.283	0.088 to 0.455	0.013	0.022
RNFL—GCIPL (*n* = 69)	0.703	0.561 to 0.840	<0.001	<0.001
RNFL—mCSA (*n* = 69)	0.220	0.002 to 0.418	0.069	0.097
GCIPL—mCSA (*n* = 69)	0.247	0.053 to 0.438	0.040	0.059

Pearson product–moment correlation; participant-bootstrap 95% confidence intervals (1500 resamples). BH-FDR adjustment was applied to the 28 correlations in this table. *n*: Number of participants included in each correlation analysis; CI: Confidence interval; BH-FDR: Benjamini–Hochberg false discovery rate; EDSS: Expanded Disability Status Scale; 9-HPT: Nine-Hole Peg Test; SDMT: Symbol Digit Modalities Test; MoCA: Montreal Cognitive Assessment; RNFL: Retinal nerve fiber layer; T25-FW: Timed 25-Foot Walk; GCIPL: Ganglion cell–inner plexiform layer; mCSA: Mean cross-sectional area.

**Table 5:** Pearson correlations of annual changes.

Pair	*n*	Pearson r	Bootstrap 95% CI	Raw *p*	BH-FDR *p*
EDSS—T25-FW	76	0.268	−0.151 to 0.550	0.019	0.206
EDSS—9-HPT	78	0.080	−0.071 to 0.322	0.486	0.772
EDSS—SDMT	74	−0.128	−0.287 to 0.071	0.277	0.772
EDSS—MoCA	76	−0.096	−0.211 to 0.053	0.409	0.772
EDSS—RNFL	69	−0.058	−0.297 to 0.257	0.634	0.866
EDSS—GCIPL	69	−0.008	−0.086 to 0.228	0.945	0.949
EDSS—mCSA	79	−0.094	−0.306 to 0.088	0.411	0.772
T25-FW—9-HPT	76	0.008	−0.214 to 0.211	0.949	0.949
T25-FW—SDMT	72	−0.176	−0.394 to 0.056	0.140	0.653
T25-FW—MoCA	73	−0.081	−0.324 to 0.146	0.496	0.772
T25-FW—RNFL	67	0.057	−0.158 to 0.293	0.647	0.866
T25-FW—GCIPL	67	−0.036	−0.160 to 0.148	0.775	0.904
T25-FW—mCSA	76	−0.151	−0.306 to 0.037	0.192	0.673
9-HPT—SDMT	73	−0.268	−0.510 to 0.019	0.022	0.206
9-HPT—MoCA	75	0.205	−0.126 to 0.478	0.078	0.543
9-HPT—RNFL	68	0.190	0.008 to 0.378	0.120	0.653
9-HPT—GCIPL	68	−0.289	−0.555 to 0.334	0.017	0.206
9-HPT—mCSA	78	−0.045	−0.245 to 0.150	0.696	0.886
SDMT—MoCA	74	−0.054	−0.338 to 0.252	0.649	0.866
SDMT—RNFL	65	−0.168	−0.396 to 0.058	0.181	0.673
SDMT—GCIPL	65	0.099	−0.166 to 0.201	0.434	0.772
SDMT—mCSA	74	−0.013	−0.219 to 0.210	0.910	0.949
MoCA—RNFL	66	−0.087	−0.297 to 0.136	0.486	0.772
MoCA—GCIPL	66	0.120	0.060 to 0.450	0.336	0.772
MoCA—mCSA	76	0.096	−0.141 to 0.296	0.408	0.772
RNFL—GCIPL	69	0.025	−0.033 to 0.406	0.837	0.937
RNFL—mCSA	69	0.036	−0.166 to 0.247	0.770	0.904
GCIPL—mCSA	69	0.122	0.094 to 0.516	0.319	0.772

Pearson product–moment correlation; participant-bootstrap 95% confidence intervals (1500 resamples). BH-FDR adjustment was applied to the 28 correlations in this table. *n*: Number of participants included in each correlation analysis; CI: Confidence interval; BH-FDR: Benjamini–Hochberg false discovery rate; EDSS: Expanded Disability Status Scale; 9-HPT: Nine-Hole Peg Test; SDMT: Symbol Digit Modalities Test; MoCA: Montreal Cognitive Assessment; RNFL: Retinal nerve fiber layer; T25-FW: Timed 25-Foot Walk; GCIPL: Ganglion cell–inner plexiform layer; mCSA: Mean cross-sectional area.

## Data Availability

The datasets analyzed in this study are available from the corresponding author upon reasonable request.

## Supplementary Materials

The following supporting information can be downloaded at: https://www.mdpi.com/article/10.3390/jcm15218491/s1, Table S1. Cervical CSA changes by anatomical level and leave-one-level-out analyses. Table S2. Partially adjusted change-score models (exploratory).
