# Supplementary material for: Longitudinal Retinal and Cervical Spinal Cord Changes in Multiple Sclerosis: Associations with Clinical, Cognitive, and Motor Measures
Source: J Clin Med. 2026 Nov 1;15(21):8491. doi: 10.3390/jcm15218491 (PMC13764167; doi:10.3390/jcm15218491)
Supplement: Supplementary file 1 [file jcm-15-08491-s001.zip › jcm-4590985-supplementary.pdf]

SUPPLEMENTARY MATERIAL

Supplementary Table S1. Cervical CSA changes by anatomical level and leave-one-level-out analyses

A. Annual change by cervical level (year 1 minus baseline)

Level	n	Median ΔCSA (mm^2)	Patients with decrease
C2–C3	79	−0.860	75/79 (94.9%)
C3–C4	79	−0.820	72/79 (91.1%)
C4–C5	79	−0.970	75/79 (94.9%)
C5–C6	79	−0.920	75/79 (94.9%)
C6–C7	79	−1.040	78/79 (98.7%)
C7–T1	79	−0.860	67/79 (84.8%)

B. Leave-one-level-out mean CSA (five-level alternative) and GCIPL change

Excluded level	Median Δmean CSA (mm^2)	Pearson r	p
None; six-level arithmetic mean	−0.912	0.112	0.360
C2–C3	−0.870	0.095	0.440
C3–C4	−0.928	0.119	0.329
C4–C5	−0.906	0.143	0.241
C5–C6	−0.920	0.048	0.698
C6–C7	−0.878	0.124	0.310
C7–T1	−0.920	0.116	0.342

Notes: Changes are year 1 minus baseline. Each level includes 79 paired measurements; each correlation with GCIPL change includes 69 participants. The original recorded mCSA remains unchanged and is used in the primary analyses (Pearson $r = 0.122$, $n = 69$, $p = 0.319$). The arithmetic mean calculated from the six source-level measurements differs from recorded mCSA in four source-verified records; therefore, the alternative five- and six-level averages are exploratory and do not replace the recorded mCSA. P values are unadjusted and descriptive. These analyses do not quantify intra-rater reliability or absolute measurement error.

Supplementary Table S2. Partially adjusted change-score models (exploratory)

Outcome	Population	n	β (ΔGCIPL)	95% CI	p
ΔmCSA	All eligible	69	0.005	−0.278 to 0.288	0.972
Δ9-HPT	All eligible	68	−0.177	−1.373 to 1.020	0.772
ΔEDSS	All eligible	69	−0.001	−0.046 to 0.045	0.979

All models include age, sex, and disease duration and use HC3 robust standard errors. Δ denotes 12 months minus baseline. These models are partially adjusted; MRI scanner and verified DMT efficacy classification were unavailable.
